# Mechanisms Underlying the Increased Plasma ACTH Levels in Chronic Psychosocially Stressed Male Mice

**DOI:** 10.1371/journal.pone.0084161

**Published:** 2013-12-23

**Authors:** Andrea M. Füchsl, Dominik Langgartner, Stefan O. Reber

**Affiliations:** 1 Department of Behavioural and Molecular Neurobiology, University of Regensburg, Regensburg, Germany; 2 Laboratory for Molecular Psychosomatics, Clinic for Psychosomatic Medicine and Psychotherapy, University Ulm, Ulm, Germany; Max Planck Institute of Psychiatry, Germany

## Abstract

Mice exposed to chronic subordinate colony housing (CSC, 19 days), an established paradigm for chronic psychosocial stress, show unaffected basal morning plasma corticosterone (CORT) concentrations, despite enlarged adrenal glands and an increased CORT response to an acute heterotypic stressor. In the present study we investigate the mechanisms underlying these phenomena at the level of the pituitary. We show that both basal and acute stressor-induced (forced swim (FS), 6 min) plasma adrenocorticotropic hormone (ACTH) concentrations, the number of total and corticotroph pituitary cells, and relative protein expression of pituitary mineralocorticoid receptor and FK506-binding protein 51 was increased in CSC compared with single-housed control (SHC) mice, while relative corticotropin releasing hormone (CRH) receptor 1 (CRH-R1) and glucocorticoid receptor protein expression was down-regulated. Relative pituitary pro-opiomelanocortin and arginine vasopressin (AVP) receptor 1b (AVPR-1b) protein expression, FS (6 min)-induced ACTH secretion in dexamethasone-blocked mice, and the number of AVP positive magnocellular and parvocellular neurons in the paraventricular hypothalamic nucleus (PVN) was unaffected following CSC. Taken together, the data of the present study indicate that 19 days of CSC result in pituitary hyperactivity, under both basal and acute heterotypic stress conditions. Although further studies have to assess this in detail, an increased number of pituitary corticotrophs together with unaffected relative pituitary AVPR-1b and decreased CRH-R1 protein expression following CSC suggests that pituitary hyperdrive is mediated by newly formed corticotrophs that are more sensitive to AVP than CRH. Moreover, our data indicate that changes in PVN AVP and negative feedback inhibition seem not to play a major role in pituitary hyperactivity following CSC.

## Introduction

The acute activation of the hypothalamus-pituitary-adrenal (HPA) axis in response to stressful stimuli represents an important mechanism to promote survival, whereas, in contrast, a prolonged increase in plasma glucocorticoid (GC) concentrations has deleterious consequences for an organism, amongst others promoting intestinal and affective disorders (for review see [1], [2], [3]). Therefore, it is beneficial for the long term health of an individual to habituate possibly fast to a prolonged and not life threatening homotypic stressor [4], [5], and to be sensitized to subsequent heterotypic and possibly dangerous challenges [6], [7], [8]. Although it is generally accepted that these habituation/sensitization phenomena do not occur if the chronic stressor is of social nature (for review see [9]), we just recently provided first evidence that this holds not true for the chronic subordinate colony housing (CSC, 19 days) paradigm, a pre-clinically validated chronic psychosocial stress paradigm relevant for human affective and somatic disorders [10], [11]. CSC compared with single-housed control (SHC) mice show unaffected basal morning plasma corticosterone (CORT) levels, despite significantly enlarged adrenals [12], and a more pronounced CORT response to an acute heterotypic stressor (elevated platform (EPF), 5 min). Interestingly, and this is in contrast to studies describing habituation to the same (homotypic) stressor and sensitization to a novel (heterotypic) stressor to be mediated mainly at the level of the pituitary gland (for review see [13]), in CSC mice adrenal mechanisms seem to play the major role. This was indicated by the fact that SHC and CSC mice did not differ in plasma adrenocorticotropic hormone (ACTH) concentrations 5 min after EPF exposure [12]. However, as basal plasma ACTH concentrations have never been assessed following CSC and as pituitary weight was increased in CSC compared with SHC mice 8 d after termination of CSC exposure [14], it can't be ruled out at this stage that pituitary mechanisms are not at least partly involved in the observed HPA axis adaptation/sensitization processes seen following 19 days of CSC exposure. To test this hypothesis was the aim of the current study.

Pituitary ACTH production and release are influenced by various stimuli, with the neuropeptides corticotropin releasing hormone (CRH) and, to a lesser extent, arginine vasopressin (AVP) from the hypothalamus representing the main ACTH secretagogues (for review see [13], [15]). CRH neurosecretory cells are located in the dorsal medial parvocellular subdivision of the paraventricular nucleus (PVN) of the hypothalamus, with about 50% of the cells coexpressing AVP [16], [17]. The axons of these neurons project to the external zone of the median eminence from where CRH and AVP are released into the pituitary portal circulation in order to drive ACTH production/secretion in/from pituitary corticotroph cells [18], [19] upon binding to CRH receptor 1 (CRH-R1) or AVP receptor 1b (AVP-R1b), respectively. ACTH, via the blood stream, subsequently reaches the adrenal cortex and induces the production and secretion of GC (for review see [15]) which, in turn, bind to both mineralocorticoid receptors (MR) and glucocorticoid receptors (GR) and mediate, amongst others, the termination of the HPA axis stress response (for review see [20]). The major sites for this GC-mediated negative feedback inhibition are the anterior pituitary gland [21], the hippocampus, and the PVN [22], [23], [24]. Upon binding of GC to the cytoplasmic MR and GR, these receptors translocate into the nucleus and either alter the transcriptional activity of GC-responsive genes by binding to specific promotor sequences (GC response elements) [25] or interact with other transcription factors [26], [27], [28]. GR functionality/sensitivity is, amongst others, regulated by its bound chaperones. The most prominent one is the heat shock protein 90 and its co-chaperone FK506-binding protein 51 (FKBP51) (for review see [29]). An increase in FKBP51, induced by GC via an ultra-short feedback loop, reduces sensitivity and in turn nuclear translocation of the receptor (for review see [29]). At the level of the pituitary, GC exert their negative feedback function by repressing the gene encoding for pro-opiomelanocortin (POMC), the precursor of ACTH [30], by directly inhibiting the release of ACTH vesicles into the periphery [31], [32] or by reducing CRH-R1 binding [33].

Therefore, to test the hypothesis that pituitary mechanisms are involved in the observed HPA axis adaptation/sensitization processes seen following CSC exposure, in the present study we examined (i) basal as well as acute stress-induced (forced swim (FS), 6 min) plasma ACTH concentrations, (ii) relative protein expression of pituitary POMC, (iii) pituitary weight and (iv) the number of total and corticotroph pituitary cells following 19 d of CSC. Moreover, we analyzed (v) relative pituitary CRH-R1 and AVP-R1b protein expression and (vi) the number of AVP positive magnocellular and parvocellular PVN neurons. Finally, to address CSC-induced changes in the negative feedback function we assessed pituitary (vii) GR and MR as well as (viii) FKBP51 relative protein expression and (ix) the ability of dexamethasone (Dex) to inhibit HPA axis activation [21] in response to an acute stressor (FS, 6 min).

## Materials and Methods

### Ethics Statement

All experimental protocols were approved by the Committee on Animal Health and Care of the local government, and conformed to international guidelines on the ethical use of animals. All efforts were made to minimize the number of animals used and their suffering.

### Animals

Male C57BL/6 mice (Charles River, Sulzfeld, Germany) weighing 19–22 g (experimental mice) were individually housed in standard polycarbonate mouse cages (16×22×14 cm) for one week before the CSC paradigm started. The male offspring (weighing 30–35 g) of high anxiety-related behavior female mice (kindly provided by Prof. Dr. R. Landgraf, Max Planck Institute of Psychiatry in Munich) and C57BL/6 male mice (Charles River, Sulzfeld, Germany) were used as dominant animals. All mice were kept under standard laboratory conditions (12 h light/dark cycle, lights on at 0600 h, 22°C, 60% humidity) and had free access to tap water and standard mouse diet.

### Experimental procedures

All experimental mice were either chronically stressed by 19-day exposure to the chronic subordinate colony housing (CSC) paradigm or single-housed for control (SHC).

On day 20 of CSC 5 sets of SHC (n = 6–13) and CSC (n = 6–20) mice were decapitated between 0800 and 1000 h and the pituitaries were removed for assessment of relative protein levels of AVPR-1b and CRH-R1 (1^st^ set), relative cytoplasmic protein levels of MR, GR, POMC and FKBP51 (2^nd^ set), ACTH immunoreactivity (3^rd^ set), overall pituitary cell number (4^th^ set), and AVP immunoreactivity in the PVN (5^th^ set). Plasma ACTH concentrations and pituitary weight was assessed in all 5 sets of SHC (n = 42) and CSC (n = 61) mice. Another set of SHC (n = 7) and CSC (n = 6) animals was used to analyze plasma ACTH levels following acute heterotypic stressor exposure (forced swim (FS), 6 min) on day 20 between 0700 and 0800 h. The last set of SHC (n = 11) and CSC (n = 12) mice was injected with either vehicle or dexamethasone (Dex) prior to FS (6 min) on day 20 to determine whether CSC affects the *in vivo* feedback response.

### Chronic subordinate colony housing (CSC)

The chronic subordinate colony housing (CSC) paradigm was conducted as described previously [10], [11], [12], [34], [35], [36], [37]. Briefly, four experimental CSC mice were housed together with a dominant male mouse for 19 consecutive days, in order to induce a chronic stressful situation. Before the CSC procedure, the future dominant males were tested for their aggressive behavior. Males that started to injure their opponents by harmful bites were not used. To avoid habituation, each dominant male was replaced by a novel dominant male at days 8 and 15. SHC mice remained undisturbed in their home cage except for change of bedding once a week. In a previous study we convincingly demonstrated that single housing is the adequate control group for the CSC paradigm, as group housing itself was shown to be stressful and to affect parameters assessed routinely in studies employing the CSC paradigm [36].

### Trunk blood sampling

On day 20, SHC and CSC mice were rapidly killed by decapitation under CO_2_ anaesthesia within 3 min after entering the animal room between 0800 and 1000 h. Trunk blood was collected in EDTA-coated tubes (Sarstedt, Nürnbrecht, Germany) on ice and centrifuged at 4°C (5000 rpm, 10 min). Per mouse, 230–240 µl plasma were collected and stored at −20°C until assayed.

### Forced swim (FS) exposure

The FS exposure on day 20 was used as acute heterotypic stressor in order to determine the acute plasma ACTH concentrations and was conducted as described previously [14]. Briefly, the FS tank consisted of an open top cylinder (25 cm height, 13 cm diameter) filled with tap water (21±1°C) to a depth of about 13 cm. Mice were immersed into the water tank for 6 min and decapitated 10 min following termination of FS exposure (under CO_2_ anaesthesia). Trunk blood was collected and stored as described above. The water was changed after every animal.

### ELISA for ACTH

Plasma samples (200 µl) were analyzed using a commercially available ELISA kit for ACTH (analytical sensitivity 0.22 pg/ml, intra-assay and inter-assay coefficients of variation ≤7.1%, IBL International, Hamburg, Germany).

### Determination of pituitary weight and pituitary cell number

After decapitation on day 20 the pituitary of each mouse was removed and weighed. For determination of the pituitary cell number, the whole pituitary was enzymatically digested in 2 ml HBSS (Life Technologies, Inc., Grand Island, NY, USA) containing Trypsin (Sigma, Deisenhofen, Germany) for 20 min at 37°C (95% O_2_, 5% CO_2_). Digestion was stopped by adding HBSS containing 10% FCS. After two washing steps with HBSS, the pituitary was triturated with a Pasteur pipette in 1 ml DMEM (DMEM/F-12, Life Technologies, Inc., Grand Island, NY, USA). After centrifugation (20°C, 10 min, 1500 rpm), cells were resuspended in DMEM/F-12 and the number of isolated cells per pituitary was assessed using a Cell Viability Analyzer (Vi-Cell™ XR, Beckman Coulter, Krefeld, Germany).

### Western Blotting

Pituitaries were removed, weighed and immediately frozen in liquid nitrogen and stored at -80°C until further preparation. For the CRH-R1 and AVP-R1b, frozen pituitaries were homogenized in ice-cold EDTA lysis buffer (0.5 mM EDTA, 250 mM NaCl, 50 mM HEPES, 0.5% Igepal, 10% complete mini protease inhibitor (Roche Diagnostics GmbH, Mannheim Germany)). For the cytoplasmic GR, MR, POMC and FKBP51, frozen pituitaries were homogenized in S1 buffer (10 mM HEPES (pH 7.9), 10 mM KCl, 1.5 mM MgCl_2_, 0.1 mM EDTA (pH 8)) supplemented with 0.5 mM dithiothreitol, 0.2 mM Na orthovanadate, 2 mM NaF and complete mini protease inhibitor (Roche Diagnostics GmbH, Mannheim, Germany). Total protein concentration was determined using a commercial kit (Bicinchoninic Acid Protein Assay Kit, Thermo Scientific, Rockford, USA).

Western Blotting was performed as described previously [11], [12], [38] using equal amounts of protein lysates (20 µg for AVP-R1b, GR, MR, FKBP51 and POMC and 40 µg for CRH-R1) and antibodies for AVP-R1b (1∶400, Santa Cruz Biotechnology, Inc., Heidelberg, Germany), CRH-R1 (1∶200, Santa Cruz Biotechnology, Inc., Heidelberg, Germany), GR (1∶700, Santa Cruz Biotechnology, Inc., Heidelberg, Germany), POMC (1∶500, Santa Cruz Biotechnology, Inc., Heidelberg, Germany), FKBP51 (1∶500, Santa Cruz Biotechnology, Inc., Heidelberg, Germany) and MR (1∶200, Santa Cruz Biotechnology, Inc., Heidelberg, Germany). After incubation with horseradish peroxidase (HRP)-conjugated donkey anti-goat secondary antibody (1∶1500 for AVP-R1b, 1∶8500 for CRH-R1, 1∶6000 for FKBP51, Santa Cruz Biotechnology, Inc., Heidelberg, Germany) or HRP-conjugated goat anti-rabbit antibody (1∶5000 for GR, 1∶1000 for POMC, 1∶2000 for MR, Cell Signaling Technology, New England Biolabs GmbH, Frankfurt am Main, Germany) immunoreactive bands were visualized with a Molecular Imager® ChemiDoc™ XRS+ system (Bio-Rad Laboratories, München, Germany) using an Enhanced chemiluminescent Western Blotting detection reagent (GE Healtcare, Freiburg, Germany). Afterwards, each membrane was stripped using Re-Blot Plus Mild Antibody Stripping Solution (Millipore GmbH, Schwalbach, Germany) and probed with primary rabbit anti-ß-Tubulin antibody (1∶1000, Cell Signaling Technology, New England Biolabs GmbH, Frankfurt am Main, Germany) as loading control for whole and cytoplasmic protein. Semiquantitative densitometric analysis of the signals was performed using Image Lab™ Software (Bio-Rad Laboratories, München, Germany). CRH-R1 (∼48 kDa), AVP-R1b (∼45–50 kDa), GR (∼86 kDa), POMC (∼27 kDa), FKBP51 (∼51 kDa) and MR (∼107 kDa) protein expression for each mouse was normalized to ß-Tubulin (∼50 kDa) expression and averaged per group. For the AVP-R1b two bands appear corresponding to the glycosylated and unglycosylated form of the receptor [39], [40], whereby both forms were considered in the semiquantitative densitometric analysis.

### Dexamethasone suppression test (DST)

SHC and CSC mice received an ip injection of either water-soluble dexamethasone phosphate (Dex, dexamethasone 21-phosphate disodium salt, Sigma, Schnelldorf, Germany) solved in sterile 0.9% saline (3 µg/100 g body weight in 100 µl) or 0.9% saline (vehicle) between 0700 and 0800 h and afterwards were returned to their home cage (SHC) or CSC cage, respectively. The dose of Dex was chosen according to another study conducted in mice [41]. Four hours following the injection, SHC and CSC were subjected to an acute heterotypic stressor (FS, 6 min, conducted as described above). 10 min following termination of FS exposure, mice were decapitated and trunk blood was collected and stored for the analysis of ACTH concentrations.

### Cryo-sectioning of pituitary tissue

After removal on day 20 of CSC pituitaries were weighed and embedded in protective freezing medium (Tissue-Tek, Sakura Finetek Europe, Zoeterwoude, The Netherlands) and stored at −80°C. Subsequently, one series of five 5-μm cryo-sections was cut using a cryostat (at -20°C) and then thaw-mounted onto pre-coated slides (SuperFrost Plus, Menzel-Gläser, Braunschweig, Germany).

### ACTH immunostaining in pituitary cryo-sections

Frozen sections were fixed with ice-cold acetone for 10 min, air-dried for 20 min and washed 2 times with PBS for 10 min. Immunohistochemical (IHC) slices were then blocked with PBS/10% normal goat serum (NGS, Biozol Diagnostica, Eching, Germany) for 20 min followed by incubation with rabbit anti-ACTH antibody (1∶200, Abcam, Cambridge, UK) in PBS/0.1% NGS overnight at 4°C. After washing 3 times with PBS for 5 min, IHC slices were incubated with the respective biotinylated anti-rabbit secondary antibody (1∶500, Vector Laboratories, Loerrach, Germany) for 30 min at RT and afterwards washed 3 times with PBS for 5 min. Respective positive cells were visualized by the use of Vectastain ABC Kit followed by Vector NovaRed Substrate Kit (Vector Laboratories, Loerrach, Germany). Finally, sections were mounted with Vectashield Mounting Medium (Vector Laboratories, Loerrach, Germany) and covered with a glass cover slip. Per slide, the area positively stained for ACTH and the visible area of the anterior pituitary were quantified in digitized images of three to six sections using Leica QWin V3 (Leica Microsystems, Wetzlar, Germany). Positively stained anterior pituitary tissue was expressed in percent of total anterior pituitary tissue. Furthermore, the number of ACTH positive pituitary cells [n] was counted in the same three to six digitized images and averaged per animal to provide individual means.

### Cryo-sectioning of brain tissue

Immediately after decapitation the brains were collected and immersed for 24 h in fixative consisting of 4% paraformaldehyde in PBS (pH 7.4). Afterwards they were cryo-protected in 30% sucrose in PBS, snap-frozen in isopentane and stored at −80°C. Serial sections (20 µm) containing the PVN, according to the mouse brain atlas [42], were cut using a cryostat (at -20°C) for AVP immunostaining and put in PBS containing 0.01% sodium azide for free floating staining.

### AVP immunostaining in brain cryo-sections

Free floating sections were incubated in PBS containing 10% NGS (Biozol Diagnostica, Eching, Germany) and 0.3% Triton-X (PBS/Triton) for 2 h at RT to block unspecific binding. This was followed by incubation with primary antibody against AVP (1∶400, p41 mouse monoclonal, which was a generous gift of Dr. Gainer, NIH, USA) in PBS/Triton/1% NGS overnight at 4°C. Afterwards, sections were washed 3 times in PBS/Triton for 5 min and incubated with the respective biotinylated goat anti-mouse antibody (1∶300, Vector Laboratories, Loerrach, Germany) for 2 h at RT and washed again 2 times with PBS/Triton for 5 min. Respective positive cells were visualized by the use of a Vectastain ABC Kit followed by DAB Peroxidase Substrate Kit (Vector Laboratories, Loerrach, Germany). Finally, the free floating sections were put on pre-coated slides (SuperFrost Plus, Menzel-Gläser, Braunschweig, Germany), mounted with Vectashield Mounting Medium (Vector Laboratories, Loerrach, Germany) and covered with a glass cover slip. Counting of positive cells was performed in digitized images using Leica QWin V3 (Leica Microsystems, Wetzlar, Germany). Parvocellular neurons in the PVN were differentiated histologically from magnocellular neurons according to their smaller size and their lower level of AVP expression [43]. According to the literature [44], the size of magnocellular neurons was approximately set to >14 µm and for parvocellular neurons <14 µm. For each animal, five to six sections, containing the regions rostral, rostral-medial, medial-caudal and caudal, were counted bilaterally. Numbers of parvocellular and magnocellular AVP positive neurons were counted within each region (1–2 sections per region) of the PVN and the average value for each mouse was calculated to provide individual means. Omission of the primary antibody resulted in no immunoreactivity, confirming specificity of the antibody used. The characteristics of the AVP antibody are further described in the original publication [45].

### Statistics

For statistical comparisons, the software package SPSS statistics (version 19.0) was used. Data of two experimental groups (SHC versus CSC) were analyzed using the parametric Student's *t*-test. The number of AVP positive neurons (factor region and factor CSC) and plasma ACTH levels following DST (factor CSC and factor stimulus) were compared using a two-way ANOVA followed by Bonferroni pairwise comparisons when appropriate. Data represent the mean + SEM. The level of significance was set at *P*<0.05.

## Results

### CSC increases basal and FS-induced plasma ACTH concentrations

**Figure 1:**
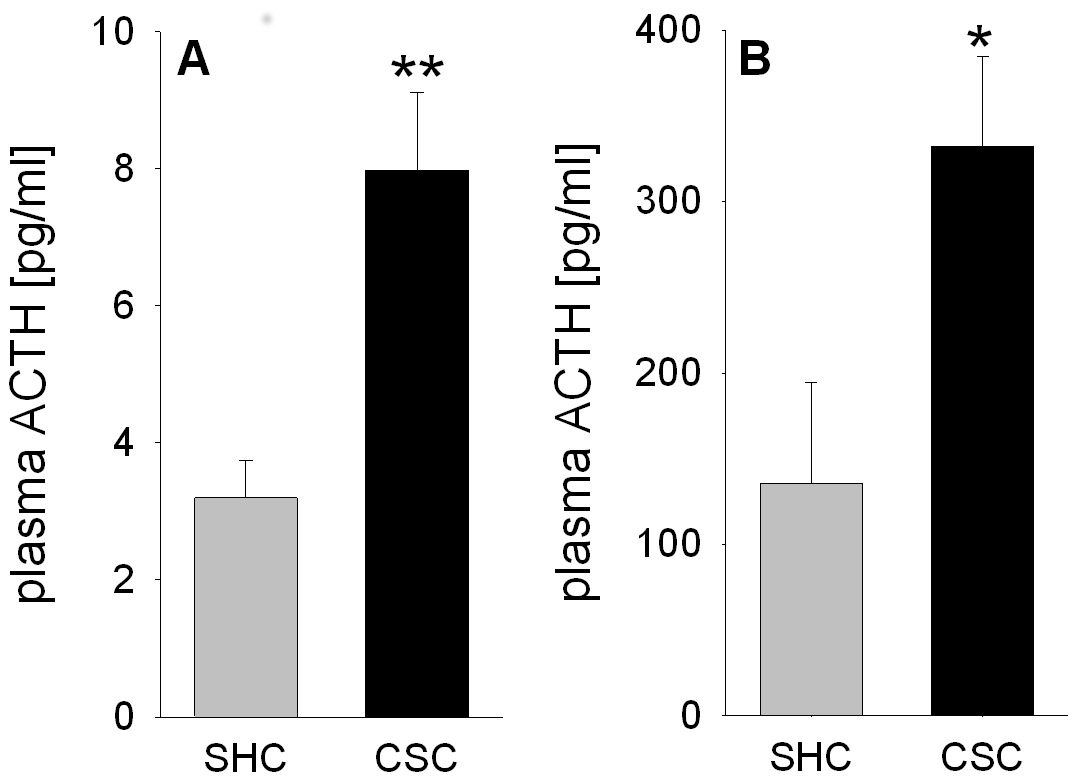
Effects of CSC on the basal morning and forced swim (FS)-induced plasma ACTH concentrations in trunk blood. Following decapitation on day 20 of CSC between 0800 and 1000[pg/ml] concentrations were determined in trunk blood of SHC (n = 42) and CSC (n = 61) mice (A). Another set of SHC (n = 7) and CSC (n = 6) mice was exposed to 6 min forced swim (FS) on day 20 of CSC and decapitated 10 min following FS exposure whereby trunk blood was collected for determination of plasma ACTH [pg/ml] concentrations (B). Grey bars represent SHC, black bars CSC mice. Data represent the mean + SEM. * represent *P*<0.05, ** represent *P*<0.01 *vs.* respective SHC mice.

Compared with SHC mice, basal morning plasma ACTH concentrations were significantly increased in CSC mice (*P* = 0.001; Fig. 1A), as were plasma ACTH concentrations 10 min following termination of FS exposure (6 min) (*P* = 0.030; Fig. 1B).

### CSC increases absolute pituitary weight as well as total and corticotroph pituitary cell number

**Figure 2:**
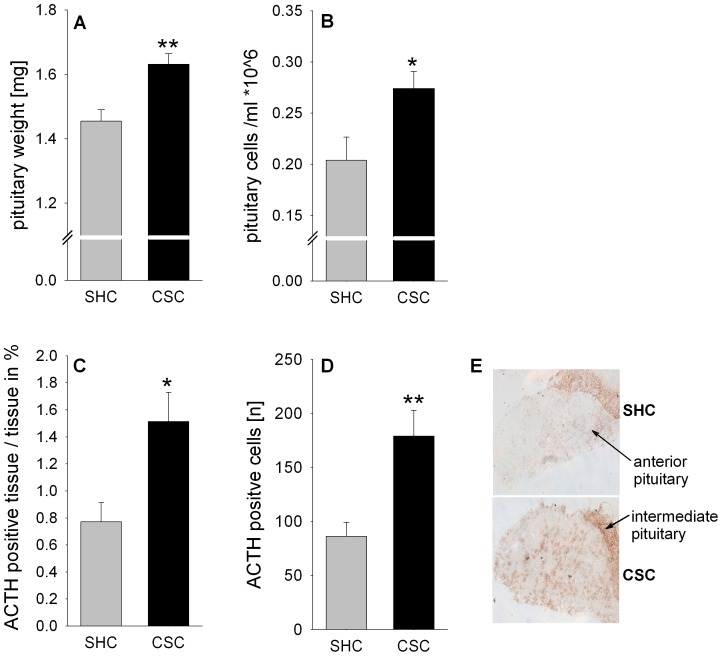
Effects of CSC on pituitary weight, pituitary cell number and the number of ACTH positive cells. Following decapitation on day 20 of CSC the pituitaries of SHC (n = 42) and CSC (n = 61) mice were removed and absolute pituitary weight [mg] was determined (A). The pituitary cells of one set of SHC (n = 6) and CSC (n = 14) mice were isolated and subsequently quantified (per ml) by means of a Cell Viability Analyzer (B). Pituitaries from another set of SHC (n = 6) and CSC (n = 6) mice were cut on a cryostat and subsequently one series of cry-sections was stained with an antibody against ACTH. Per animal, three to six different sections were analyzed and averaged for the percentage of ACTH positive anterior pituitary tissue in relation to the total anterior pituitary tissue (C). Moreover, the number of ACTH positive pituitary cells [n] was counted in the same three to six digitized images and averaged per animal to provide individual means (D). Grey bars represent SHC, black bars CSC mice. Data represent the mean + SEM. * represent *P*<0.05, ** represent *P*<0.01 *vs.* respective SHC mice. (E) Representative images of cryo-sections stained with antibody against ACTH showing anterior and intermediate pituitary.

Absolute pituitary weight (*P* = 0.001, Fig. 2A), the number of isolated pituitary cells (*P* = 0.030; Fig. 2B), the percentage (in relation to anterior pituitary tissue) of ACTH positive tissue (*P* = 0.019; Fig. 2C/E), as well as the number of ACTH positive corticotroph cells (*P* = 0.009; Fig. 2D/E) was significantly increased in CSC compared with SHC mice.

### CSC does neither affect POMC nor AVP-R1b, but decreases CRH-R1 relative protein expression in the pituitary

**Figure 3:**
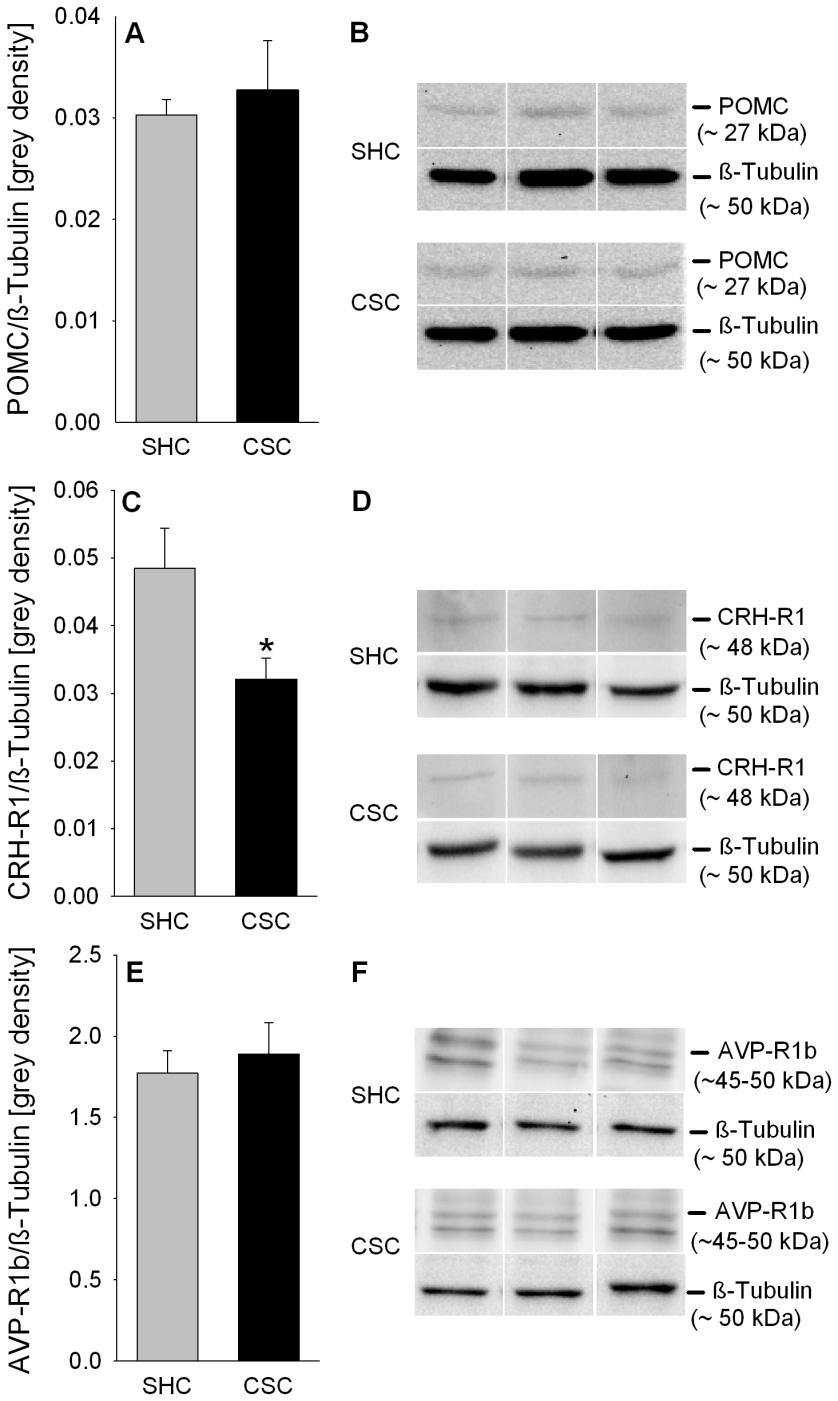
Effects of CSC on relative POMC, CRH-R1 and AVP-R1b protein expression in the pituitary. Following decapitation on day 20 pituitaries of both SHC and CSC mice were removed. Afterwards, protein was extracted from the pituitaries either for determination of relative POMC (SHC: n = 13, CSC: n = 13; A), CRH-R1 (SHC: n = 9, CSC: n = 12; C), and AVPR-1b (SHC: n = 8, CSC: n = 20; E) protein expression [grey density], all normalized to the loading control ß-Tubulin. Grey bars represent SHC, black bars CSC mice. Data represent the mean + SEM. *represents *P*<0.05 *vs.* respective SHC mice. Representative images of bands detected for POMC (∼27 kDa; B), CRH-R1 (∼48 kDa; D) and AVP-R1b (∼45–50 kDa; F) and respective loading control ß-Tubulin (∼50 kDa; B/D/F) are shown for SHC and CSC mice.

Statistical analysis revealed no differences between CSC and SHC mice regarding POMC (Fig. 3A/B) and pituitary AVP-R1b (Fig. 3E/F) relative protein expression. However, relative pituitary CRH-R1 protein expression was significantly reduced in CSC compared with SHC mice (*P* = 0.029; Fig. 3C/D).

### CSC does not affect the number of AVP positive neurons in the PVN

**Figure 4:**
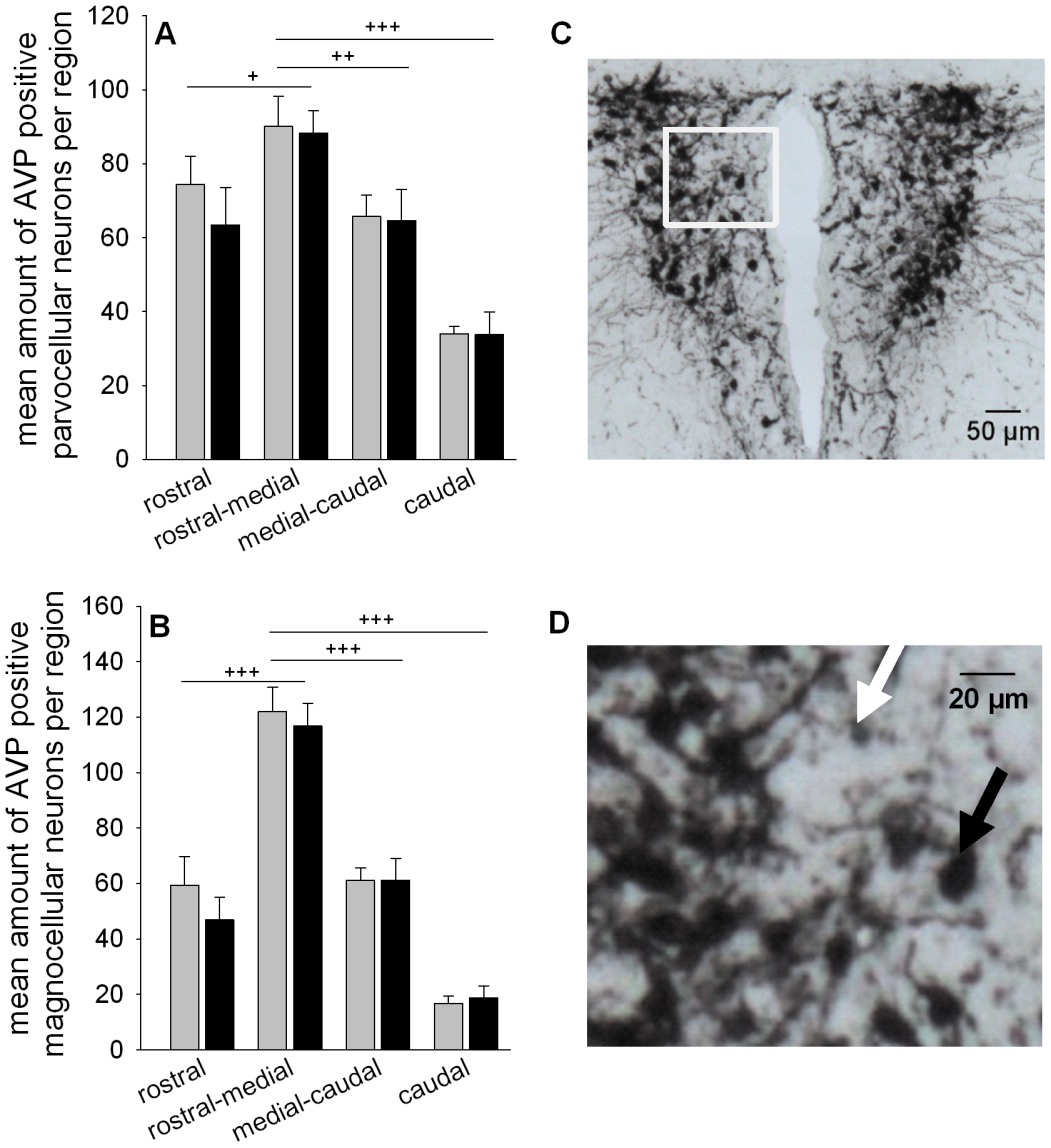
Effects of CSC on the number of AVP positive neurons in the PVN. Following decapitation on day 20 of CSC brains were removed and immersed in fixative. One series of cryo-sections (5–7 sections per animal), containing the regions rostral, rostral-medial, medial-caudal and caudal was stained for AVP employing a free-floating approach. Number of parvocellular (A) and magnocellular (B) AVP positive neurons were counted within each region (1–2 sections per region) of the PVN and averaged per animal for SHC (n = 8 per region) and CSC (n = 7–8 per region). Grey bars represent SHC, black bars CSC mice. Data represent the mean + SEM. ^+^ represent *P*<0.05, ^++^ represent *P*<0.01, ^+++^ represent *P*<0.001 *vs.* rostral-medial region. (C) Representative image of cryo-section stained for AVP in the PVN (magnification: 5×; scale bar 50 µm). (D) Higher magnification (400×) of the magnocellular (black arrow) and parvocellular (white arrow) AVP positive neurons depicted in the white frame of Figure 4C (scale bar 20 µm).

Two-way ANOVA considering the factors region (rostral, rostral-medial, medial-caudal, caudal) as well as CSC only revealed significant effects for factor region in parvocellular (F_1,55_ = 19.886; *P*<0.001; Fig. 4A) and magnocellular (F_1,55_ = 64.861; *P*<0.001; Fig. 4B) AVP positive neurons. In detail, the number of AVP positive parvocellular and magnocellular neurons was significantly increased within the rostral-medial part of the PVN compared to the rostral (parvocellular: *P* = 0.037; magnocellular: *P*<0.001), medial-caudal (parvocellular: *P* = 0.008; magnocellular: *P*<0.001) and caudal part (parvocellular and magnocellular: *P*<0.001).

### CSC decreases GR and increases MR relative protein expression in the pituitary

**Figure 5:**
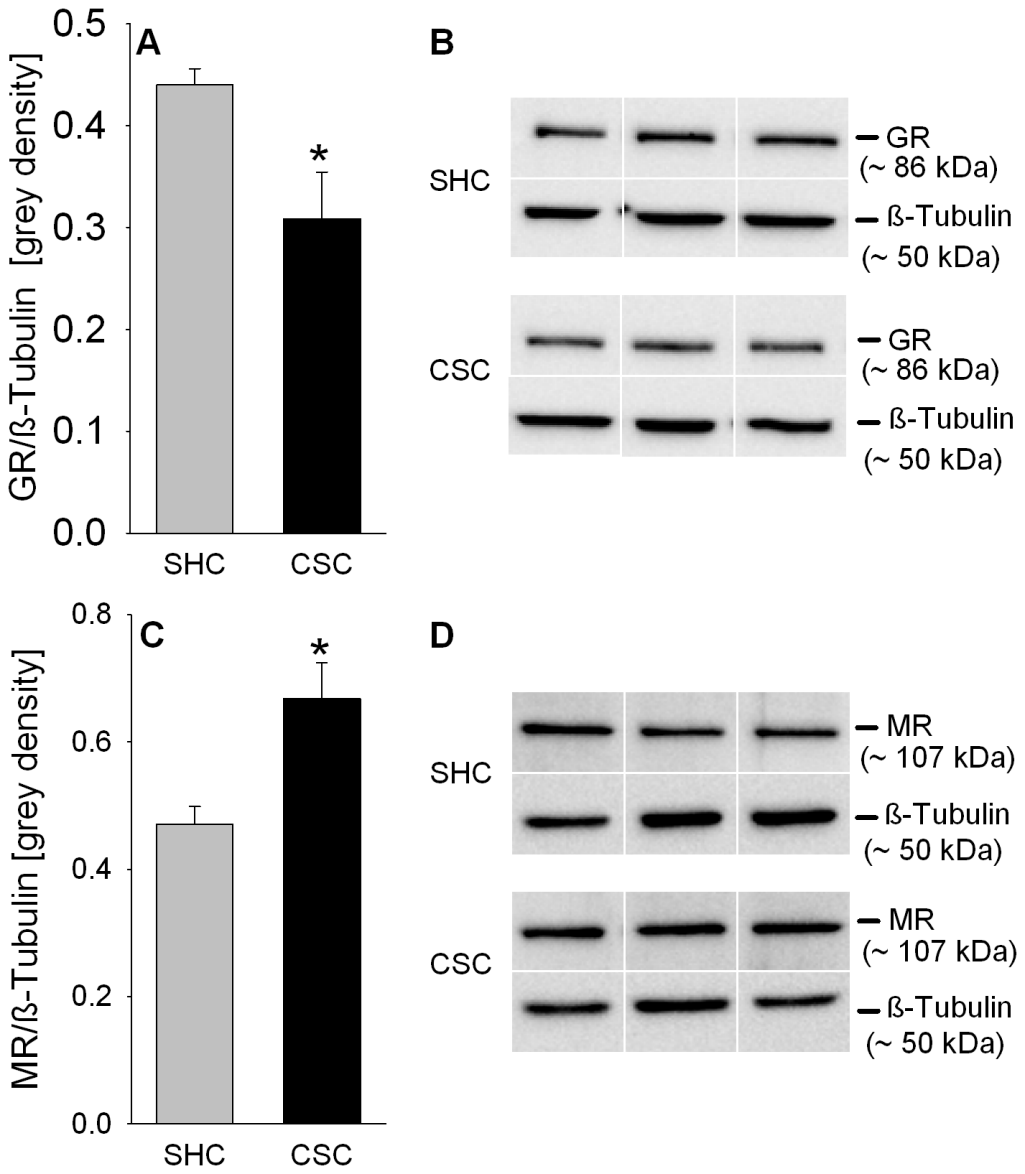
Effects of CSC on relative GR and MR protein expression in the pituitary. Following decapitation on day 20 pituitaries were removed and protein was extracted from the pituitaries of SHC (GR: n = 11; MR: n = 8) and CSC (GR: n = 10; MR: n = 7) mice for determination of relative GR and MR protein expression [grey density] normalized to the loading control ß-Tubulin (A/C). Grey bars represent SHC, black bars CSC mice. Data represent the mean + SEM. * represents *P*<0.05 *vs.* respective SHC mice. Representative images of bands detected for GR (∼86 kDa; B) or MR (∼107 kDa; D) and respective loading control ß-Tubulin (∼50 kDa; B/D) are shown for SHC and CSC mice.

Statistical analysis revealed that under basal conditions relative cytoplasmic GR protein expression (*P* = 0.011; Fig. 5A/B) was decreased while respective MR expression (*P* = 0.014; Fig. 5C/D) was increased in CSC compared with SHC mice.

### CSC increases plasma ACTH response to FS exposure in vehicle-injected but not in Dex-blocked mice

**Figure 6:**
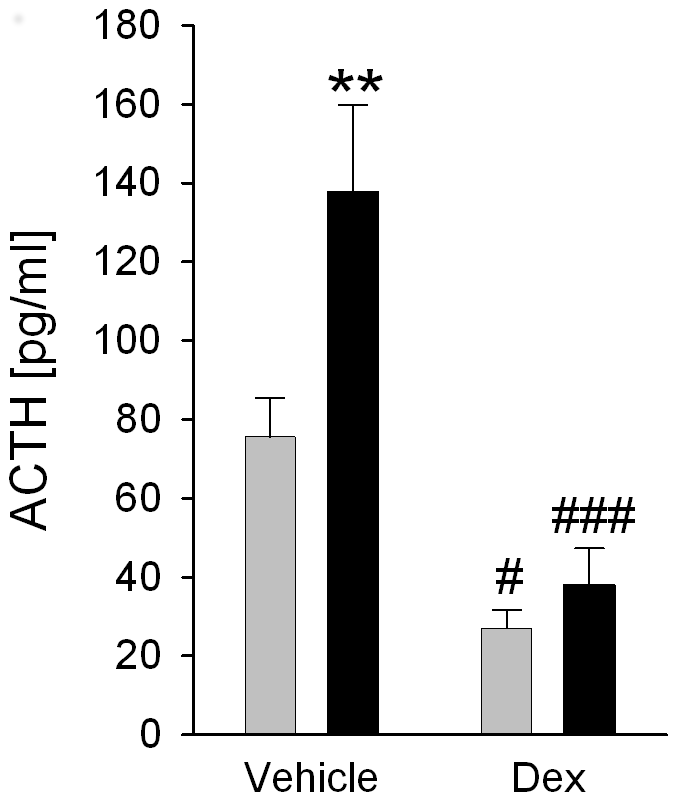
Effects of CSC on the plasma ACTH concentrations in trunk blood following DST. On day 20 of CSC, between 0700 and 0800(30 µg/kg, ip). Four hours later all animals were exposed to forced swim (FS) for 6 min. 10 min after termination of the acute stressor exposure all mice were decapitated and trunk blood was collected for determination of plasma ACTH [pg/ml] (SHC: n = 11; CSC: n = 12). Grey bars represent SHC, black bars CSC mice. Data represent the mean + SEM. ** represent *P*<0.01 *vs.* respective SHC mice; # represent *P*<0.05, ### represent *P*<0.001 *vs.* respective vehicle group.

Plasma ACTH release during FS was dependent on both factor CSC (F_1,42_ = 7.18; *P* = 0.010) and factor treatment (F_1,42_ = 29.74; *P*<0.001; Fig. 6). In detail, ACTH response to FS was increased in vehicle-injected CSC compared with respective SHC mice (*P = *0.002), an effect that was absent in Dex-blocked mice. Moreover, FS-induced ACTH release was significantly lower in Dex-blocked SHC (*P = *0.018) and CSC (*P<*0.001) mice compared with respective vehicle-injected mice.

### CSC increases relative FKBP51 protein expression in the pituitary

**Figure 7:**
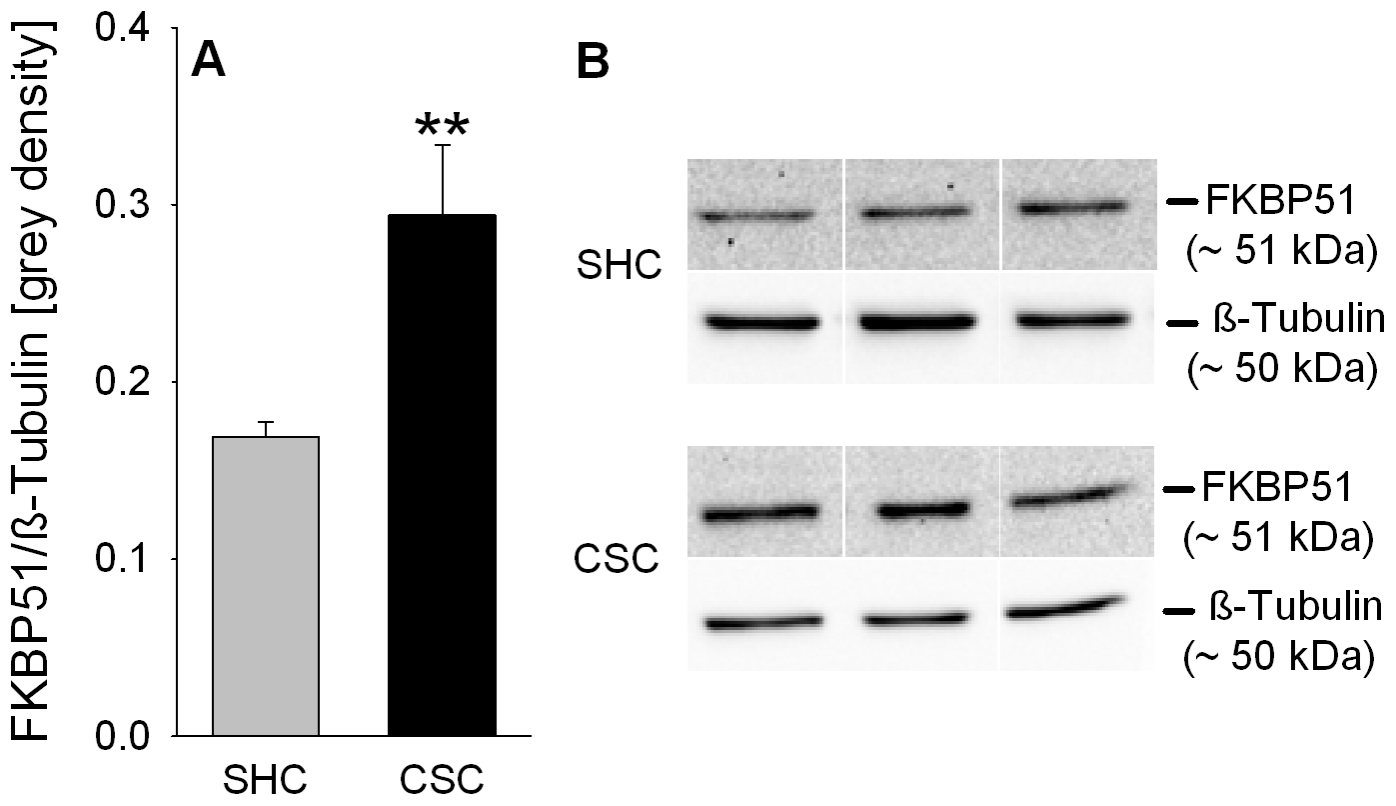
Effects of CSC on relative FKBP51 protein expression in the pituitary. Following decapitation on day 20 of CSC pituitaries of both SHC (n = 13) and CSC (n = 13) mice were removed for protein extraction and subsequent determination of relative FKBP51 protein expression [grey density] normalized to the loading control ß-Tubulin (A). Grey bars represent SHC, black bars CSC mice. Data represent the mean + SEM. ** represents *P*<0.01 *vs.* respective SHC mice. (B) Representative images of bands detected for FKBP51 (∼51 kDa) and the loading control ß-Tubulin (∼50 kDa) are shown for SHC and CSC mice.

Relative pituitary FKBP51 protein expression was significantly increased in CSC compared with SHC mice (*P* = 0.006; Fig. 7A/B).

## Discussion

In the present study we reveal, and this is in accordance with our initial hypothesis, that CSC not only affects the adrenals but also causes alterations at higher HPA axis levels, i.e. the pituitary gland. In detail, CSC results in pituitary hyperactivity, under both basal and acute heterotypic stress conditions, mediated most likely by corticotroph cell hyperplasia and, thus, the increased availability of fully functional - relative pituitary POMC protein expression was comparable between CSC and SHC - ACTH producing and secreting pituitary cells. Although further studies have to clarify this in detail, unaffected relative pituitary AVPR-1b and decreased CRH-R1 protein expression in this context imply that these newly formed corticotrophs are shifted in their sensitivity from CRH to AVP, providing support for the idea that during conditions of prolonged/chronic stress AVP becomes the main pituitary ACTH secretagogue. However, as the number of AVP positive parvocellular PVN neurons was comparable between CSC and SHC mice, an increased PVN AVP output – as suggested by other studies dealing with repeated/chronic stressor exposure – seems not to further promote pituitary AVP stimulation and, thus, to contribute to the increased ACTH drive in CSC mice. Moreover, the contributing role of changes in the negative feedback inhibition to the increased basal and stress-induced ACTH secretion in CSC mice seems to be negligible, at least at the level of the pituitary, as DST indicated a fully functional feedback system. The latter finding clearly indicates that a decrease in pituitary cytoplasmic GR protein expression cannot generally be interpreted as impairment of negative feedback function, a finding per se of significance for the field of stress research.

19 days of CSC exposure in the present study resulted in increased basal plasma morning ACTH concentrations, even though previous studies reported comparable plasma CORT concentrations between CSC and SHC mice at that time of the day [10], [12]. Together with the decreased *in vitro* adrenal ACTH responsiveness in CSC mice [10], [12], this clearly supports the idea of a developing adrenal ACTH insensitivity during/following CSC; probably to prevent basal hypercorticism as a consequence of the CSC-induced increase in adrenal mass [10], [11] and basal plasma ACTH concentrations.

As this increased basal ACTH drive following CSC is in contrast to the desensitization of the ACTH response described during repeated homotypic stressor exposures, like e.g. repeated immobilization [6], [46], the mechanisms underlying this phenomenon were in the focus of the present study. Interestingly, exposure to a subsequent heterotypic stressor, i.e. 6-min of FS, in CSC compared with SHC mice resulted in an exaggerated ACTH response, suggesting not only pituitary hyper-activity, but also hyper-reactivity. This facilitated response to a novel heterotypic challenge following chronic stressor exposure is in line with other studies [6], [47] but in contrast to own recent data showing similar ACTH levels in SHC and CSC mice 5 min following termination of a 5-min EPF exposure [12]. The most likely explanation for this discrepancy simply lies in the experimental design and the intensity of the acute stressor applied. In contrast to the present study where 10 min elapsed between acute stressor termination and killing, CSC and SHC mice in the Uschold-Schmidt study (2012) were killed only 5 min after heterotypic stressor exposure. Moreover, given that in CSC mice plasma ACTH levels were about 10-fold higher following FS (∼300 pg/ml) than EPF (∼25 pg/ml) exposure, FS in the present study represents a much more severe acute heterotypic challenge than EPF. The latter might be due to the fact that EPF exposure represents an exclusively emotional stressor, while FS exposure additionally holds a strong physical component [48]. Thus, it is likely that an increased ACTH response to acute heterotypic stressors following CSC needs a certain time and/or stressor severity to develop. In general this supports the idea that the process of HPA axis adaptation during CSC is mainly achieved at the level of the adrenal gland [12], whereas adrenal [11], [12] and pituitary mechanisms are combined to realize the concept of HPA axis sensitization to acute heterotypic stressors in CSC mice.

As main factor contributing to the increased pituitary activity/re-activity following CSC, we in the present study identified pituitary enlargement, paralleled by an increased number of overall and, most importantly, corticotroph pituitary cells. The latter was indicated by an increased percentage of ACTH positive pituitary tissue as well as number of ACTH positive cells in the anterior pituitary of CSC compared with SHC mice. Corticotrophs represent one out of five major cell types in the anterior pituitary (corticotrophs, thyrotropes, gonadotropes, somatotropes and lactotropes) (for review see [49]), characterized by the production and secretion of ACTH. Pituitary hyperplasia on day 20 of CSC, as seen in the present study, is in line with an elevated pituitary weight previously described 8 days after termination of CSC exposure [14], indicating that CSC-induced changes in HPA axis parameters are reliable and long lasting. Importantly, an unaffected relative pituitary protein expression of POMC – the precursor molecule of ACTH - together with an increased number of corticotrophs indicates that newly formed corticotrophs during CSC are fully functional and, thus, allow a CSC mouse to overall produce and secrete increased amounts of ACTH. To investigate whether increased pituitary activity/re-activity following CSC is additionally driven by an increased stimulatory input from the PVN, we, in a next step, quantified relative pituitary protein expression of the two main ACTH secretagogue receptors, namely AVPR-1b and CRH-R1. As relative AVPR-1b protein expression was unaffected and CRH-R1 protein expression even decreased in CSC compared with SHC pituitaries, an increased secretagogue receptor-mediated pituitary input, at least at the first glance, seems not to play a major role in increased pituitary drive following CSC exposure. However, when considering that the number of corticotrophs was increased in CSC compared with SHC mice these relative protein expression data suggest that newly formed corticotrophs during CSC only express the AVPR-1b and not the CRH-R1 and, thus, are shifted in their sensitivity from CRH to AVP. Although, this would be in accordance to studies suggesting AVP to become an important ACTH secretagogue during conditions of prolonged/chronic stress [43], conclusive insight needs to be revealed in future studies quantifying for instance ACTH release during *in vitro* pituitary stimulation with different doses of CRH, AVP and a combination of both as well as coexpression of proliferation markers with ACTH, AVPR-1b, and CRH-R1 protein employing immunohistochemistry.

An increased pituitary input might also be due to an exaggerated ACTH secretagogue production in the PVN. Based on our above discussed pituitary protein findings we in the present study quantified the number of AVP positive neurons in the PVN of CSC and SHC mice employing immunohistochemistry and differentiating between magnocellular and parvocellular neurons according to their staining intensity, size, and localization within the PVN [43], [44]. Support for this approach was also provided by the down-regulated AVP mRNA expression that we previously reported in the PVN of CSC compared with SHC mice [34]. Decreased mRNA levels, at least in the supraoptic nucleus, have been interpreted to represent an increased mRNA turn over and, thus, to indicate increased AVP protein synthesis [50]. However, comparable numbers of magno- and parvocellular AVP containing PVN neurons between CSC and SHC mice suggested that at least the PVN AVP system is not further - in addition to possible AVPR-1b-mediated effects - promoting pituitary AVP stimulation and, thus, contributing to the increased ACTH drive in CSC mice. Importantly, our finding of magnocellular and parvocellular AVP neurons being localized mainly in the mid part of the PVN is in accordance to what is described in another study in mice [44], supporting our experimental approach and the way we quantified AVP positive PVN neurons.

As increased basal and acute stress-induced ACTH concentrations in CSC mice might be mediated, or at least exaggerated, by a decreased negative feedback inhibition, another aim of this study was to assess GR expression at the level of the pituitary and Dex suppression during acute heterotypic stressor exposure. Interestingly, and in line with this hypothesis, an increased pituitary weight in patients with depression and psychosis [51], [52] is often associated with a decreased negative feedback inhibition (for review see [53], [54]). Alterations in the negative feedback function following 19 days of CSC exposure are further not unlikely as former studies revealed a decreased GC sensitivity in both splenocytes [10] and T helper 2 cells from peripheral lymph nodes [35] of CSC compared with SHC mice. However, although a down-regulated relative pituitary GR protein expression in CSC compared with SHC mice at the first glance supports a compromised feedback function, performance of the DST following CSC clearly indicated that under *in vivo* conditions the negative feedback function is, if at all, increased following CSC. The latter was indicated by a comparable or even increased Dex suppression of acute stressor (10 min after a 6-min FS exposure)-induced ACTH release in CSC compared with SHC mice. In detail, while vehicle-injected CSC mice showed increased plasma ACTH levels following FS compared with respective SHC mice – an effect that is in line with our data obtained in respective un-injected mice (see Fig. 1) - Dex treatment reduced the plasma ACTH response to FS in SHC and, to an even larger extent, in CSC mice.

Interestingly, an increased GR sensitivity and/or GR nuclear translocation in CSC compared with SHC mice - suggested by the lower relative cytoplasmic pituitary GR protein expression but unaffected Dex suppression - is supported by an elevated relative pituitary FKBP51 protein expression following CSC. FKBP51 expression is induced by GC via an ultra-short feedback loop (for review see [29]) and, thus, can be interpreted as marker for the potential of GC to bind to GR, subsequent GR translocation, and DNA binding. However, given that FKBP51 reduces sensitivity and in turn nuclear translocation of GR (for review see [29]) it might also be interpreted as additional indication for a reduced GR signaling, in line with the reduced GR protein expression [55]. Moreover, in mice overexpressing MR in the hippocampus it was shown, that at least under stressful conditions, MR can partly reconstitute negative feedback under GR deficiency [56]. As the pituitary also expresses both, GR and MR [57], [58], [59], we finally analyzed pituitary relative cytoplasmic MR protein expression and found it increased in CSC compared with SHC mice. However, whether in CSC mice the decreased relative cytoplasmic GR expression is compensated by an increased GR translocation or by the presence of increased MR has to be addressed in detail in further studies.

Taken together, in the present study we show that 19 days of CSC result in pituitary hyperactivity, under both basal and acute heterotypic stress conditions, mediated most likely by corticotroph cell hyperplasia and, thus, the increased availability of fully functional ACTH producing and secreting pituitary cells. Unaffected relative pituitary AVPR-1b and decreased CRH-R1 protein expression following CSC further suggests that these newly formed corticotrophs are more sensitive to AVP than CRH. Moreover our data support the idea that an increased stimulatory PVN AVP output as well as a decreased negative feedback inhibition do not play a major role in pituitary hyperactivity following CSC exposure. Together with previous findings, the process of HPA axis adaptation during CSC seems to be mainly achieved at the level of the adrenal gland, whereas adrenal and pituitary mechanisms act synergistically to realize the concept of HPA axis sensitization to acute heterotypic stressors.
